# Sub-gluteal ligation of the internal pudendal vein for management of veno-occlusive erectile dysfunction (Shaeer’s Vein Ligation–I): the cadaveric study

**DOI:** 10.1038/s41443-024-00846-1

**Published:** 2024-02-29

**Authors:** Osama Shaeer, Ehab A. A. El-Shaarawy, Hossam Y. Emam, Islam Elsisi, Eslam Sokar, Kamal Shaeer

**Affiliations:** 1 Department of Andrology, Kasr El Aini Faculty of Medicine, Cairo University, Cairo, Egypt; 2 The Middle East Society for Sexual Medicine, Cairo, Egypt; 3 Department of Anatomy and Embryology, Kasr El Aini Faculty of Medicine, Cairo University, Cairo, Egypt

**Keywords:** Surgery, Urethra

## Abstract

Vein ligation for veno-occlusive erectile dysfunction is being abandoned due to the recurrence rate. Among the reasons for failure is inability to ligate the deep system of veins; the internal pudendal vein. The vein exits the pelvis in the gluteal region, from the lesser sciatic foramen to the greater sciatic foramen, coursing over the ischial spine and sacro-spinous ligament, under the gluteus maximus. This work aims to verify feasibility of the first surgical procedure to ligate the internal pudendal vein through the gluteal approach. This cadaveric study involved five formalin-fixed cadavers. A surface anatomical landmark was designed to identify the ischial spine, at the intersection of two lines: a vertical line from posterior superior iliac spine to ischial tuberosity, and a horizontal line extending from sacro-coccygeal joint, laterally. An incision is cut encompassing the target point. Subcutaneous fat is dissected down to the gluteus maximus, which is split along the direction of its fibers. The vein can be found crossing over the ischial spine. “Shaeer’s Vein Ligation – I” appears to be surgically feasible. A protocol for a surgical study is registered at clinicaltrials.gov, and is open for participation.

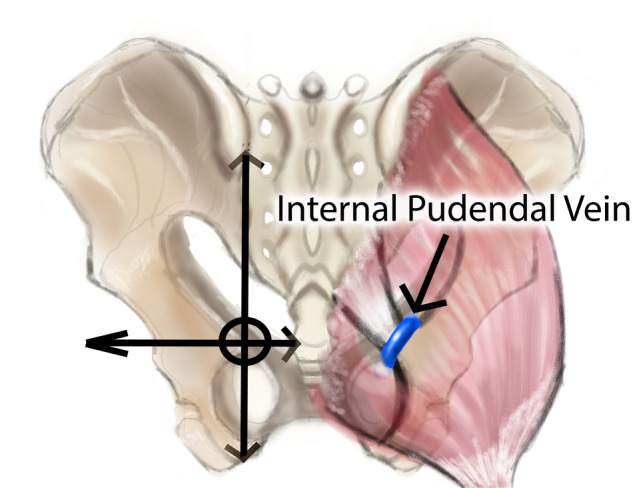

## Introduction

Erectile dysfunction (ED) is a global health problem. Prevalence of ED is estimated to be 13.1–71.2% of males, variable with age and comorbidities [1]. Etiology is often multifactorial. Potential underlying etiologies can be psychogenic or organic. With a perspective on penile hemodynamics which drive the process of erection, organic etiologies often translate into either arteriogenic ED (arterial stenosis) or veno-occlusive dysfunction (VOD). VOD is when there is failure of penile corporal rigidity despite adequate arterial influx, with a high venous efflux. This is demonstrated in penile duplex studies by consistently normal peak systolic velocities and high end-diastolic velocities (EDV > 5 cm/sec) [2].

In case of failure of medical therapy for ED, the main surgical option is penile prosthesis implantation (PPI), with patient satisfaction rates exceeding 85% [3]. Yet, PPI can be costly, and prone to complications (though rare), such as prosthetic infection and mechanical failure [4, 5]. Vein ligation surgery had been described as early as 1897 [6] and has since been modified and re-examined. This mainly refers to deep dorsal vein ligation (DVL) [7] or embolization [8]. Currently, this surgery is rarely performed considering the failure rates and recurrence rates [9]. The low or short-lived efficacy of DVL is multifactorial, including the pathogenesis behind it being endothelial and smooth muscle damage rather than excessive venous outflow [10], which is the consensus at the time of performing the present work.

Regardless the pathogenesis, rapid efflux of blood through certain veins occurs, resulting in loss of rigidity. If effective occlusion of those veins could be achieved, this would possibly enhance rigidity. Hence the use of vacuum suction with basal compression in a subset of patients. Otherwise, the only option for many such cases refractory to treatment comes down to PPI. One of the proposed causes for the low efficacy and short-lived sustainability of vein ligation is missing some venous outlets at the time of surgery [11, 12], such as the deep system of veins. The penis is drained by 3 systems of veins, the superficial, intermediate, and deep. The deep system is formed of the crural and cavernosal veins. Both drain mainly into the internal pudendal vein (IPV) [13], though may communicate with the periprostatic (retropubic) plexus [11]. IPV has so far been surgically inaccessible.

The IPV courses through Alcock’s canal, along with the internal pudendal artery and the pudendal nerve (pudendal neurovascular bundle). They emerge from the pelvis posteriorly, through the lesser sciatic foramen which is bounded superiorly by the ischial spine (IS) and sacrospinous ligament (SSL). The neurovascular bundle crosses over the IS and SSL, covered by the gluteus maximus muscle (GM). The bundle then dips back into the pelvis through the greater sciatic foramen.

This work is a cadaveric study describing Shaeer’s Vein Ligation-I (SVL-I), the first surgical technique for ligation of IPV as it courses superficial to the IS, deep to the GM, through a gluteal incision. To our knowledge, this is the first report of such an approach.

## Subjects and methods

**Fig. 1:**
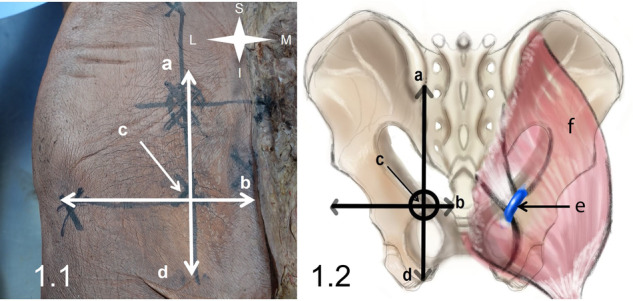
Surface anatomy landmarks for the internal pudendal vein. **a**: The posterior superior iliac spine. **b**: Sacro-coccygeal joint. **c**: Surface anatomy landmark for the ischial spine. **d**: Ischial tuberosity. **e**: Internal pudendal vein. **f**: Gluteus maximus. The ischial spine at the intersection between two lines:—Line (**a**–**d**): extending from the posterior superior iliac spine (**a**) to the ischial tuberosity (**d**).—Line b: extending laterally from the sacro-coccygeal joint (**b**).—The internal pudendal vein is on or immediately medial to the ischial spine (**c**).

Approval from the ethical committee was obtained before commencing the study (approval number MD-282-2023). The study was conducted at the Department of Anatomy and Embryology, Kasr Al Aini Faculty of Medicine, Cairo University, Egypt. SVL-I was performed unilaterally in five formalin-fixed cadavers, with the gluteal region intact. Surface anatomical landmarks were used to identify the IS, at the intersection between two lines: a vertical line bridging the posterior superior iliac spine to the ischial tuberosity, and a horizontal line extending from the sacro-coccygeal joint, laterally (Fig. 1). This point is the surface anatomy of the IS. The IPV courses on or just medial to the IS, deep to the GM.

A vertical incision encompassing the target point for the IS was cut, lateral to the anal cleft, and extended laterally in a hockey-stick fashion as needed. Subcutaneous fat was dissected down to the GM, which was split along the direction of its fibers. The IPV was identified deep to GM, coursing between the pudendal nerve medially and the pudendal artery laterally. IPV was ligated. The split fibers of GM were approximated. Subcutaneous fat and skin were sutured closed. Video of the surgical technique is available at the Video Journal of Sexual Medicine [14].

## Results

In five cadavers, IPV was successfully identified and ligated at the target point. Figure 1 shows the anatomical landmarks which lead to the IS and subsequently the IPV. Those anatomical landmarks were verified to point to the IPV in all cases.

**Fig. 2:**
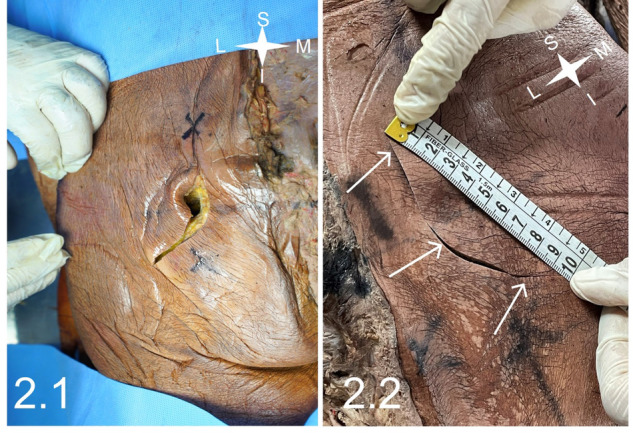
Skin incision. A hockey stick skin incision encompasses the surface anatomy landmark for the ischial spine (2.1) and its length in 2.2 (arrows).

Figure 2 shows the skin incision in the gluteal region. Extension of the incision in a hockey-stick fashion was required in all cases (Fig. 2.1). The mean length of the incision was 9.3 ± 0.6 cm (range: 9–10 cm) (9–10 cm) (Fig. 2.2), varying with the bulk of tissue down to the IPV.

**Fig. 3:**
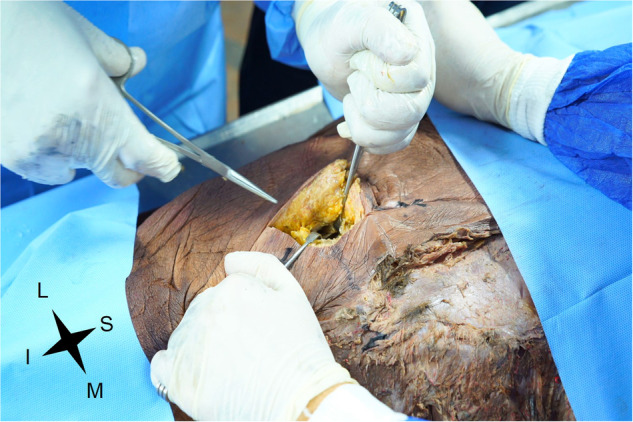
Subcutaneous fat dissection.

Figure 3 shows the dissection of the subcutaneous fat.

**Fig. 4:**
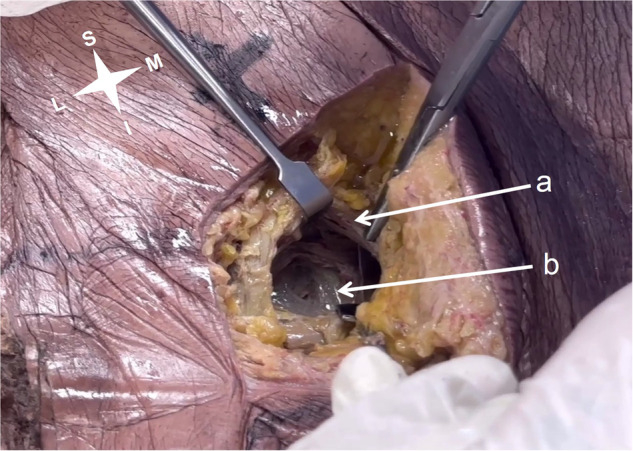
Muscle splitting. Splitting the gluteus maximus along its fibers (**a**) showing the inferior gluteal neurovascular bundle (**b**).

Figure 4 shows the splitting of the GM fibers along their direction (coursing downwards and laterally) (a), and the inferior gluteal neurovascular bundle within the muscle (b).

**Fig. 5:**
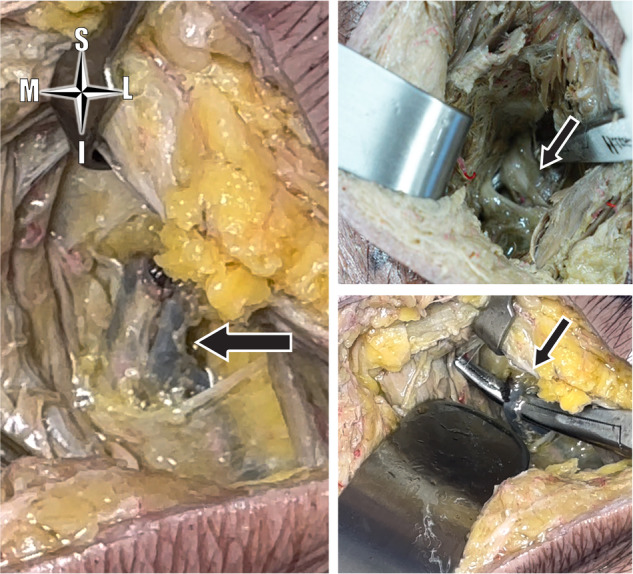
Identification of the internal pudendal vein. The internal pudendal vein (arrow) identified deep to the gluteus maximus, within fat.

**Fig. 6:**
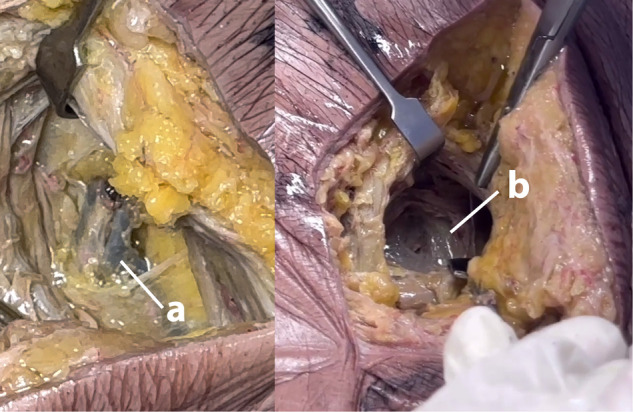
Avoiding false identification. The internal pudendal neurovascular bundle surrounded by fat (**a**), as opposed to the inferior gluteal neurovascular bundle within the GM bulk (**b**).

**Fig. 7:**
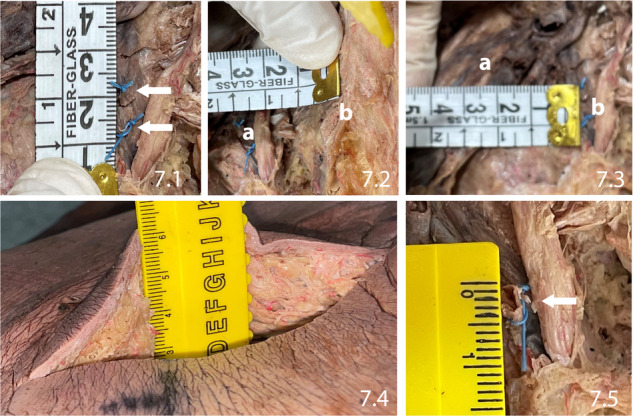
Showing the different landmarks of internal pudendal nerve (IPV). 7.1: Ligated and cut internal pudendal vein (arrows). 7.2: The distance between internal pudendal vein (**a**) and lateral wall of sacrum (**b**). 7.3: The distance between sciatic nerve (**a**) and internal pudendal vein (**b**). 7.4: The depth from skin surface to reach the internal pudendal vein. 7.5: The internal pudendal vein diameter in cut-section.

In all cases, IPV width was 0.5 cm, IPV length was 2.7 ± 1 cm (1.8–4 cm), and the distance from IPV to sciatic nerve was 5.1 ± 1 cm (4–6 cm). IPV was retrieved at a depth of 5.2 ± 1.6 cm (3–8 cm) from the skin surface Figs. 5–7. The procedure in the first cadaver took approximately 40 min time. This came down to 20–30 min in the subsequent four cadavers. Although the formalin-fixed cadavers were firm, there was no noticeable difficulty in reaching the IPV. This should be even easier with frozen-thawed cadavers (which were not available), and most importantly with live surgery.

## Discussion

### Venous surgery for ED

In the 1980’s and 90’s, DVL and its variants were extensively tried. This included classic DVL [7], DVL and spongiolysis [15], with further modifications down the road such as excision of the deep dorsal vein [16], venous stripping surgery and refined venous stripping [17], embolization through the DDV [18] and crural ligation [19]. Leak-afflicted systems can be identified by cavernosography or computed tomography cavernosography for patient selection and surgical planning [20]. Deep system leak could be indentified in 94.2% of cases suffering VOD, using computed tomography cavernosography [21]. However, the root of the deep system of veins (IPV) remained surgically inaccessible.

### Shaeer’s Vein ligation—I (SVL-I)

“SVL-I” is to our knowledge the first technique for ligation of the IPV. The surgical technique has been established in this cadaveric study. SVL-I can be performed unilaterally or bilaterally, with or without retrograde embolization, and with or without dorsal vein occlusion. This should be dictated by cavernosography. Efficacy and sustainability are yet to be tested. A theoretical risk for SVL-I may be priapism. Patients selected for SVL-I should be those refractory to medical treatment for ED, and are destined for a penile implant. If refractory priapism actually occurs, the patient will have PPI performed promptly.

### Surgical tips

#### Avoiding false identification of the IPV

In the first case, we initially miss-identified a longitudinal facia-covered structure within the deeper bulk of the gluteus maximus as being the IPV. We decided to explore further down to confirm our finding. We eventually retrieved the IPV at a deeper level, surrounded by fat and directly related to bone. We verified the correct identification of the IPV by identifying the adjacent artery and two nerves (internal pudendal artery, pudendal nerve, and nerve to obturator internus), by identifying the glistening white striated fibers of the SSL below IPV, and by dissecting along the same plane to the sciatic nerve laterally, and to the sacral bone medially. We believe that the initially miss-identified structure could have been ramifications of the inferior gluteal neurovascular bundle within the muscle tissue; pure muscle tissue with no fat around. Accordingly, identification of IPV should only be confirmed deep to the GM muscle fibers, within fat, not within muscle, and with the sacral bone medial Fig. 6. The fore mentioned exploration was not required in subsequent cadavers, and will not be required in live surgery. This exploration in the first cadaver is the reason why it took 40 min, compared to an average of 25 min for the subsequent cases.

#### Expected depth for dissection down to IPV

The depth at which the IPV was encountered ranged from 3 to 8 cm, variable with the bulk of gluteal fat and muscle. In patients with obesity, a surgical headlight should be helpful, particularly if extent of the skin incision is to be confined to the shortest possible length.

#### Avoiding injury of adjacent structures

##### The inferior gluteal nerve (IGN)

The IGN is a primarily motor nerve. It typically courses underneath the piriformis muscle before emerging at its caudal border medial to the sciatic nerve and dividing into several branches, that enter the inferior third of the GM on its deep surface [22]. The IGN can be found at the junction of a line connecting the most prominent lateral border of the greater trochanter horizontally, with a perpendicular vertical line from the ischial tuberosity [22]. Median distance from IGN to ischial spine was 28.5 mm (range, 6-53 mm) [23].

Cutaneous branches of the IGN may occur in the lower outer quadrant of the GM and travel in an inferosuperior or inferolateral direction. These branches had a mean distance of 12.5 cm from the midline [24].

Injury to the nerve is mostly iatrogenic and leads to an altered gait pattern known as gluteus maximus ‘lurch.’ Injury is more likely when a muscle-splitting incision is across the GM rather than along the muscle fibers [25]. A triangular-shaped anatomic area that contains the IGN was described, formed by connecting the following points: posterior inferior iliac spine, ischial tuberosity and the greater trochanter. This triangle can further be divided into lower triangle and the upper triangle. The latter is the “danger zone” that contains the IGN and its branches [26]. Yet, procedures involving severing the GM are commonly performed, an example being the posterior approach for hip arthroplasty [27]. Injuries without nerve transection can be managed conservatively [28].

The main trunk of IGN enters the gluteus maximus 5–6 cm medial to the tip of the greater trochanter well away from our field. It courses down medial and adjacent to the sciatic nerve which we measured to be 5.1 ± 1 cm lateral to the IPV. The cutaneous branches emerge in the lateral gluteal region, again away from our field. SVL-I is performed on the medial perimeter of the danger zone [26] rather than within. Therefore -in our opinion- injury of the main trunk of IGN far-fetched.

In order to avoid injury of smaller branches of the IGN, we recommend that GM splitting is maintained medially, dissection and retraction of the muscle should be with blunt instruments, and along the direction of its fibers rather than across them. We also recommend that once fat appears beneath the muscle, finger dissection/sweeping should be employed to split the muscle further, thus avoiding injury of underlying vessels and of the IGN by the surgical instruments.

When suturing the gluteus maximus closed, we recommend sparing the deeper layer of the muscle where the nerve courses, and confining suturing to the intermediate and superficial layers of muscle bulk. Pre-marking the anatomical landmark of the IGN [22] and the “danger zone” [26] on the gluteal skin can be employed if needed.

##### Sciatic nerve

The sciatic nerve is 5.1 ± 1 cm lateral to our anatomical target point and the IPV. Therefore there should be no risk of injury to the sciatic nerve, Sciatic nerve cannot be mistaken for any other structure, considering its large caliber, one of the largest nerves in the lower limb. IPV emerges from the pelvis and dips back in, for a short length. This is in contrast to the sciatic nerve which runs freely down the lower limb.

##### Internal pudendal artery, pudendal nerve, and nerve to obturator internus

Identification, dissection and separation of the IPV from the adjacent artery and nerves was relatively easy and did not require optical magnification, considering its caliber (0.5 cm). We found IPV to be the most lateral of the internal pudendal neurovascular bundle structures, though there may be anatomical variations. We expect identifications of the IPV to be even easier in live surgery compared to formalin-fixed cadavers. However, if needed, identification in live surgery can be further facilitated by induction of artificial erection, thereby augmenting arterial pulsations and vein engorgement. Working with cavernosography under C-arm is another possible addition.

#### Concealment of the incision

We performed a hockey-stick incision in this cadaveric study. This was required considering the tissue stiffness encountered with formalin-fixed cadavers. Average length was 9.3 ± 0.6 cm. In live surgery, we recommend starting with a shorter vertical incision, and only extend length and adopt the hockey-stick form if needed, particularly with obese patients.

The more medial the incision is, the more concealed it will be, within the anal cleft. In one case, the incision was more medial than planned. The sacral bone was therefore encountered once dissection was beyond the gluteus maximus. This was simply resolved by sliding laterally, and the IPV was identified with ease. So it appears that a more medial incision is feasible, serving both concealment and protection against injury to the branches of the IGN.

Finally, despite the debate over pathogenesis of VOD and the controversial prior results for venous surgery, vein occlusion has some logic behind it, if only its results could be improved. In the current era, with a plethora of emerging novel solutions for ED (such as regenerative therapies), there is always a need for innovation. High ligation of the IPV in the gluteal region (SVL–I) appears to be surgically feasible. It is yet to be seen whether SVL-I is efficacious and if the effect is sustained, in clinical trials. A multi-center clinical study has been registered at clinicaltrials.gov, and is open for participation from interested centers [29].

## Data Availability

Data are available within the published article.
